# Jingmenviruses: Ubiquitous, understudied, segmented flavi-like viruses

**DOI:** 10.3389/fmicb.2022.997058

**Published:** 2022-10-10

**Authors:** Agathe M. G. Colmant, Rémi N. Charrel, Bruno Coutard

**Affiliations:** Unité des Virus Émergents (UVE: Aix-Marseille Univ-IRD 190-Inserm 1207), Marseille, France

**Keywords:** jingmenvirus, arbovirus, tick-borne disease, segmented flavivirus, emerging virus

## Abstract

Jingmenviruses are a group of viruses identified recently, in 2014, and currently classified by the International Committee on Taxonomy of Viruses as unclassified *Flaviviridae*. These viruses closely related to flaviviruses are unique due to the segmented nature of their genome. The prototype jingmenvirus, Jingmen tick virus (JMTV), was discovered in *Rhipicephalus microplus* ticks collected from China in 2010. Jingmenviruses genomes are composed of four to five segments, encoding for up to seven structural proteins and two non-structural proteins, both of which display strong similarities with flaviviral non-structural proteins (NS2B/NS3 and NS5). Jingmenviruses are currently separated into two phylogenetic clades. One clade includes tick- and vertebrate-associated jingmenviruses, which have been detected in ticks and mosquitoes, as well as in humans, cattle, monkeys, bats, rodents, sheep, and tortoises. In addition to these molecular and serological detections, over a hundred human patients tested positive for jingmenviruses after developing febrile illness and flu-like symptoms in China and Serbia. The second phylogenetic clade includes insect-associated jingmenvirus sequences, which have been detected in a wide range of insect species, as well as in crustaceans, plants, and fungi. In addition to being found in various types of hosts, jingmenviruses are endemic, as they have been detected in a wide range of environments, all over the world. Taken together, all of these elements show that jingmenviruses correspond exactly to the definition of emerging viruses at risk of causing a pandemic, since they are already endemic, have a close association with arthropods, are found in animals in close contact with humans, and have caused sporadic cases of febrile illness in multiple patients. Despite these arguments, the vast majority of published data is from metagenomics studies and many aspects of jingmenvirus replication remain to be elucidated, such as their tropism, cycle of transmission, structure, and mechanisms of replication and restriction or epidemiology. It is therefore crucial to prioritize jingmenvirus research in the years to come, to be prepared for their emergence as human or veterinary pathogens.

## Introduction

According to the International Committee on Taxonomy of Viruses, the genus *Flavivirus*, family *Flaviviridae*, includes 53 officially identified species and 38 related unclassified viruses (mammalian tick-borne, mosquito-borne, insect-specific flaviviruses, viruses with no known arthropod vector, segmented flavi-like viruses) (International Committee on Taxonomy of Viruses [ICTV], 2022). Flaviviruses are found worldwide and can be responsible for significant human and veterinary diseases (Pierson and Diamond, 2020). Examples include the mosquito-transmitted dengue, West Nile, Japanese encephalitis, yellow fever, Zika viruses, or the tick-transmitted tick-borne encephalitis virus, which infect hundreds of millions of individuals each year (Pierson and Diamond, 2020). Most of the identified flaviviruses to date are either mosquito- or tick-borne in which case their replication cycle alternates between an arthropod vector and a vertebrate host (International Committee on Taxonomy of Viruses [ICTV], 2022). However, some flaviviruses have been found to be insect-specific, while others have no known vector associations (International Committee on Taxonomy of Viruses [ICTV], 2022).

The genome of flaviviruses is composed of an 11 kilobase-long single molecule of positive single-stranded RNA (+ssRNA), with no polyadenylation at the 3 prime end, and is encapsidated in an enveloped ∼45 nm icosahedral virus particle (Westaway et al., 1985). The genome contains an open reading frame encoding for a single polyprotein co- and post-translationally cleaved into three structural and seven non-structural proteins (Westaway et al., 1985). Non-structural proteins of interest here are NS5, the most conserved, which contains the RNA-dependent RNA polymerase (RdRp) and methyltransferase (MTase) domains, and NS2B/NS3 which forms the viral protease while NS3 also has a helicase domain (Westaway et al., 1985).

Qin et al. (2014) published the discovery and identification of a flavi-like virus with a segmented genome. Jingmen tick virus (JMTV) was identified as a flavi-like virus due to the high level of similarity of two of its segment sequences with the NS5 and NS2B/NS3 proteins of flaviviruses. Since this discovery, and due to the development and more affordable access to high throughput next-generation sequencing, JMTV sequences have been detected all over the world, alongside multiple other species of segmented flavi-like viruses. This review details what has been found on segmented flavi-like viruses, and why research on these viruses should be a high priority, in particular due to their high risk of emergence as pathogens.

## Tick- and vertebrate-associated jingmenviruses

### Jingmen tick virus

**FIGURE 1:**
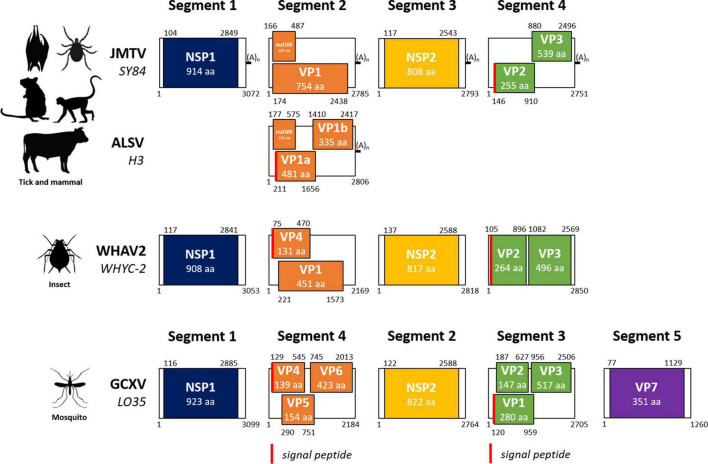
Genome organization of jingmenviruses. The layout of this figure is modified from Kobayashi et al. (2021). JMTV SY84, Jingmen tick virus strain SY84 (NC_024113-NC_024117); ALSV H3, Alongshan virus strain H3 (MH158415–MH158418); WHAV2 WHYC-2, Wuhan aphid virus 2 strain WHYC-2 (KR902725–KR902728); GCXV LO35, Guaico Culex virus strain LO35 (KM461666-KM461670). (A)_n_, polyadenylation. NSP1, non-structural protein 1, RNA-dependent RNA polymerase and methyltransferase domains; VP, virus (structural) protein; nuORF, open reading frame identified by Kholodilov et al. (2020); NSP2, non-structural protein 2, serine protease and helicase domains. Red bar: signal peptide. The genome organization of JMTV and ALSV are representative of tick- and mammal-associated jingmenviruses; WHAV2 represents insect-associated jingmenviruses; GCXV represents mosquito-associated jingmenviruses.

**FIGURE 2:**
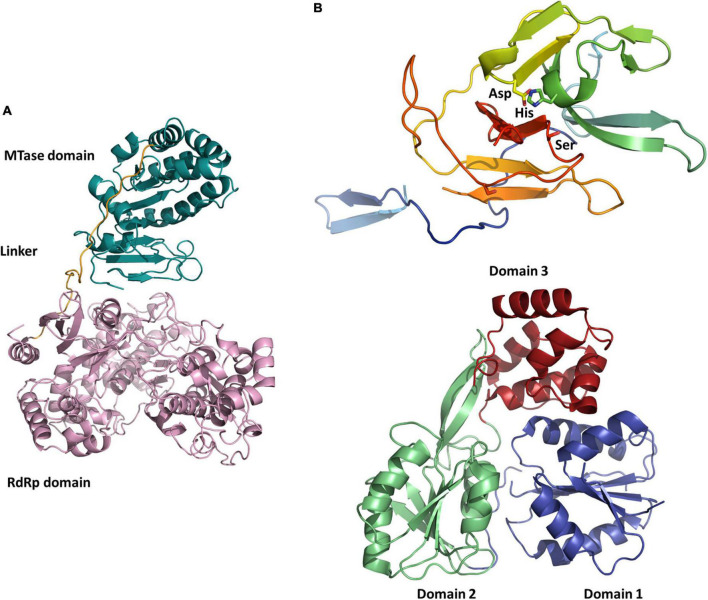
Structures of NSP1 and NSP2 functional domains putatively involved in the replication. **(A)** Cartoon representation of the NSP1 MTase (turquoise) and RdRp (pink) domains of Guaico Culex virus (homology model). **(B)** Cartoon representation of the NSP2 protease domain of Jingmen tick virus (homology model) with the canonical Asp-His-Ser catalytic triad (top) and NSP2 helicase domain of Alongshan virus (PDB code 6M40, from X-ray crystallography data in Gao et al., 2020) organized in subdomains 1 (blue), 2 (green), and 3 (ochre) (bottom). All representations were prepared using PyMOL (Schrödinger).

Segmented flaviviruses, i.e., flavi-like viruses with segmented genomes, were first described by Qin et al. (2014) as Jingmen tick virus (JMTV) was identified in a pool of *Rhipicephalus microplus* ticks collected in the Jingmen region of Hubei province in China in 2010 (Qin et al., 2014). The newly identified virus was shown to have a (+ssRNA) genome composed of four 3 prime polyadenylated segments, one bicistronic and three monocistronic, with 5 prime (GCAAGUGCA) and 3 prime (GGCAAGUGC) termini conserved sequences across segments (Qin et al., 2014). To date, no study has addressed the existence or nature of an RNA cap in 5 prime as in flaviviruses, but the genome contains an MTase domain, which is known to be involved in the cap synthesis for flaviviruses (Liu et al., 2010). Indeed, the first segment encodes for NSP1, a flavivirus NS5-like protein with putative RNA-dependent RNA polymerase (RdRp) and methyltransferase domains (Figures 1, 2; Qin et al., 2014). Segment 3 encodes for NSP2, which shares similarities with the NS2B/NS3 flavivirus protein complex, namely, transmembrane regions, serine protease, and helicase domains (Qin et al., 2014). Cartoon representations of the structure of functional domains of both non-structural proteins are shown in Figure 2 for three representative viruses, including JMTV. The other two segments encode for VP1 (segment 2) and VP2-3 (segment 4) which are thought to be structural proteins, likely to be the putative envelope, capsid, and membrane proteins, respectively (Qin et al., 2014). An additional open reading frame (ORF) overlapping with VP1 coding sequence at the start of segment 2 has been proposed for JMTV and related viruses (nuORF, or VP4), which would encode for a small membrane protein (Kholodilov et al., 2020). The overall length of the JMTV genome is 11,401 nucleotides, similar to that of flaviviruses (Qin et al., 2014).

A partial sequence from Brazilian *R. microplus* was identified in 2013 as part of a flaviviral genome (termed Mogiana tick virus – MGTV), prior to the characterization of JMTV (Maruyama et al., 2014). The sequences were later found to share 93–97% amino acid identity with JMTV, suggesting these are strains of the same virus, but the segmented nature of its genome had not been identified at the time (Maruyama et al., 2014; Villa et al., 2017).

**FIGURE 3:**
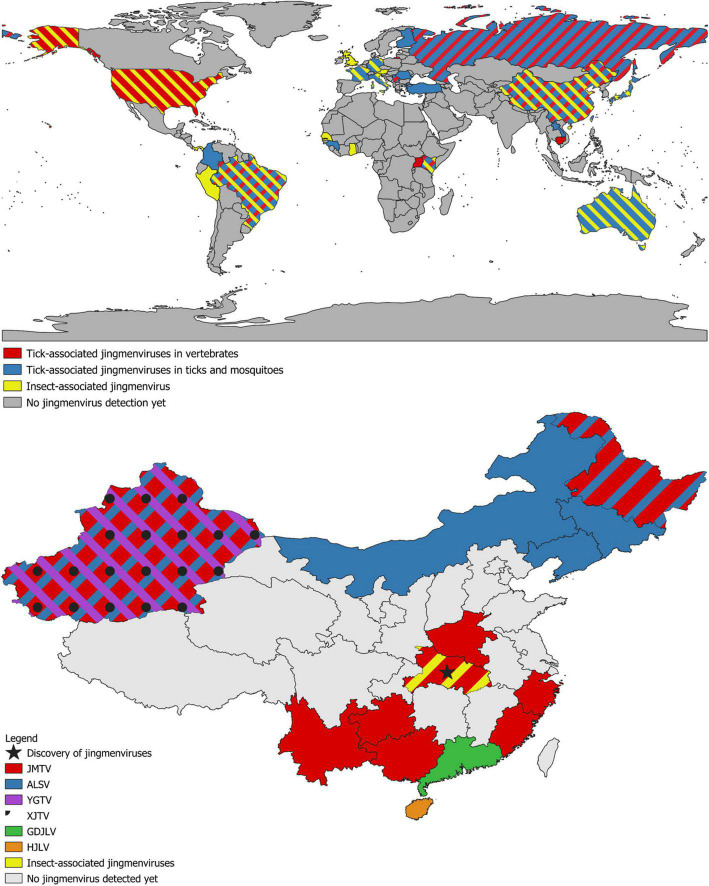
**(Top)** World map of detections of jingmenvirus sequences. Countries in which tick-associated jingmenviruses have been detected in vertebrates contain red; countries in which tick-associated jingmenviruses have been detected in ticks and mosquitoes contain blue; countries in which insect-associated jingmenviruses have been detected contain yellow. **(Bottom)** Map of detections of jingmenvirus sequences in Chinese provinces. The province where jingmenviruses were first discovered is highlighted with a black star. Provinces in which Jingmen tick virus (JMTV) has been detected contain red; provinces in which Alongshan virus (ALSV) has been detected contain blue; provinces in which Yanggou tick virus (YGTV) has been detected contain purple; provinces in which Xinjiang tick virus (XJTV) has been detected contain black spots; provinces in which Guangdong jingmen-like virus (GDJLV) has been detected contain green; provinces in which Hainan jingmen-like virus (HJLV) has been detected contain orange; provinces in which insect-associated jingmenviruses have been detected contain yellow. All other provinces are in gray. These maps were drawn using the QGIS software and open-source geographic data.

Since the discovery of JMTV, sequences of JMTV strains have been detected from samples originating from four continents: Asia (China, Lao PDR, Japan), Europe (Turkey, Italy, Kosovo, Romania, Russia), the Americas (Brazil, Trinidad and Tobago, French Antilles, Colombia) and Africa (Uganda, Guinea, Kenya) (see Table 1 and Figure 3). To date, the species in which JMTV RNA has been detected most commonly is the tick *R. microplus.* In fact, JMTV was found in high prevalence in *R. microplus* from China (53–63%), Brazil (25–67%), Trinidad and Tobago (6–46%), and the French Antilles (24–77%) over the years (Maruyama et al., 2014; Qin et al., 2014; Souza et al., 2018; Pascoal et al., 2019; Sameroff et al., 2019; Temmam et al., 2019; Gondard et al., 2020; Guo J. J. et al., 2020; Kobayashi et al., 2021; Xu et al., 2021). However, the nature of the samples containing JMTV RNA extends beyond this species and includes 25 additional tick species from several genera, *Rhipicephalus, Amblyomma, Dermacentor, Haemaphysalis, Hyalomma*, and *Ixodes* (Maruyama et al., 2014; Qin et al., 2014; Souza et al., 2018; Dinçer et al., 2019, 2022; Jia et al., 2019; Meng et al., 2019; Pascoal et al., 2019; Sameroff et al., 2019; Temmam et al., 2019; Gondard et al., 2020; Guo J. J. et al., 2020; Gómez et al., 2020; Ternovoi et al., 2020b; Xu et al., 2020, 2021; Bratuleanu et al., 2021; Li et al., 2021; Shi et al., 2021; Ogola et al., 2022), as well as two mosquito species (Qin et al., 2014; Parry et al., 2021). The local prevalence of JMTV RNA in tick species such as *Hae. longicornis, Hae. campanulata, D. nuttalli, Hae. hystricis* in different regions of China can be as high as 11–55, 75, 75, and 46% respectively (Qin et al., 2014; Meng et al., 2019; Guo J. J. et al., 2020; Xu et al., 2021). While these numbers are calculated either with individual tick numbers (minimum infection rate) or estimated from pool numbers, it is nonetheless clear that JMTV can be found in high prevalence in ticks with a widespread geographical distribution, particularly in *R. microplus*. This invasive tick species is considered the most important cattle tick in the world since it can transmit a range of diseases and has spread from Asia to a range of tropical and subtropical regions of Africa, America, and Australasia (Estrada-Peña et al., 2006a,b; Barré and Uilenberg, 2010). The distribution of *R. microplus* is predicted to expand even further and reach Western Europe due to climate change (Marques et al., 2020). An example of this unstoppable spread is North America, where an eradication program took place over 40 years in the first half of the 20th century, which could not prevent the recent reintroduction of *R. microplus* or its predicted spread throughout this continent (Giles et al., 2014).

**TABLE 1:** Tick- and vertebrate-associated jingmenviruses RNA detections.

Virus	Host species	Host type	Location	Date	Genbank number	References
JMTV	*R. microplus, R. sanguineus, Hae. longcornis, Hae. campanulata, Hae. flava, I. sinensis, I. granulatus*	Tick	China	2010	KJ001547–KJ001582, KJ001584–KJ001634	Qin et al., 2014
JMTV	*Armigeres* sp.	Mosquito	China	2010	KJ001583	
JMTV	*Bos* sp.	Cattle	China	2010	UNP	
JMTV^1^	*R. microplus*	Tick	Brazil	2006	JQ289026–JQ289041, JX390985–JX390986, KY523073–KY523074, HS586608–HS586670	Maruyama et al., 2014; Villa et al., 2017
JMTV	*Piliocolobus rufomitratus*	Monkey	Uganda	2012	KX377513–KX377516	Ladner et al., 2016
JMTV	*Homo sapiens*	Human	Kosovo	2013–2015	MH133313–MH133324	Emmerich et al., 2018)
JMTV	*Bos taurus*	Cattle	Brazil	2016	MH155885–MH155889	Souza et al., 2018
JMTV	*R. microplus*	Tick	Brazil	2015–2016	MH155890–MH155908	
JMTV^1^	*R. microplus*	Tick	Brazil	2016	MH033852–MH033854	Pascoal et al., 2019
JMTV	*I. persulcatus, Hae. longicornis, Hae. concinna, D. nuttalli*	Tick	China	2015	MG880118–MG880119, UNP	Meng et al., 2019
JMTV^2^	*A. javanense, I. persulcatus, D. silvarum, Hae. concinna*	Tick	China	2016	MG703252–MG703255, UNP	Jia et al., 2019
JMTV	*Homo sapiens*	Human	China	2010–2018	UNP	
JMTV	*R. microplus*	Tick	Trinidad and Tobago	2017	MN025512–MN025520	Sameroff et al., 2019
JMTV^3^	*R. microplus, A. variegatum*	Tick	French Antilles	2014–2015	MN095523–MN095526	Temmam et al., 2019
JMTV	*A. testudinarium*	Tick	Lao PDR	UNP	MN095527–MN095530	
JMTV	*R. bursa, R. turanicus, Hya. marginatum, Hae. parva, R. sanguineus, Hae. inermis*	Tick	Turkey	2013–2018	MN486256–MN486271, MN308066–MN308078	Dinçer et al., 2019
JMTV^4^	*R. geigyi*	Tick	Guinea	2017	MH678723–MH678730, MK673133–MK673136	Ternovoi et al., 2020b
JMTV	*Hae. hystricis, R. microplus*	Tick	China	2012	MK721553–MK721576	Guo J. J. et al., 2020
JMTV	*Bubalus bubalis*	Cattle	China	2014	MK721577–MK721596	
JMTV	*Rat. tanezumi, Rat. norvegicus, Apo. agragius*	Rodent	China	2016	MK721597–MK721612	
JMTV	*Myo. davidii, Myo. laniger, Min. fuliginosus, Nyc. noctula, Pip. abramus, Ept. andersoni, Rhi. sinicus, Myo. siligorensis, Rhi. pusillus, Rhi. pearsonii, Rhi. ferrumequinum*	Bat	China	2016	MK721613–MK721680	
JMTV^1^	*R. microplus*	Tick	Colombia	2017	MK683452–MK683457	Gómez et al., 2020
JMTV^1^	*A. testudinarium*	Tick	China	2016	MT080097–MT080100	Xu et al., 2020
JMTV	*Mic. arvalis, Mic. gregalis, Apo. uralensis, Cri. migratorius, Rho. opimus, Mus musculus, Mer. tamariscinus, Mer. libycus*	Rodent	China	2016	MK174230–MK174257, MN369292–MN369308, UNP	Yu et al., 2020
JMTV	*R. microplus, Hae. longicornis*	Tick	China	2019	MW721954–MW722053, MZ964982–MZ964985	Xu et al., 2021
JMTV	*R. bursa*	Tick	Romania	2019	MW561147–MW561150	Bratuleanu et al., 2021
JMTV	*R. microplus*	Tick	China	2017	MH814977–MH814980	Shi et al., 2021
JMTV^5^	*Ae. albopictus*	Mosquito	Italy	2017	BK059426–BK059429	Parry et al., 2021
JMTV	*A. testudinarium*	Tick	Japan	2013–2020	LC628148–LC628179	Kobayashi et al., 2021
JMTV	*R. appendiculatus, A. sparsum, A. nuttalli, A. species, Hya. truncatum, R. evertsi evertsi*	Tick	Kenya	2019	ON158818–ON158867, ON186499–ON186526	Ogola et al., 2022
JMTV	*Stigmochelys pardalis*	Tortoise	Kenya	2019	ON158817	
JMTV	*R. bursa, R. turanicus*	Tick	Turkey	2020	MZ852764–MZ852766	Dinçer et al., 2022
JMTV	*I. persulcatus*	Tick	China	2016	UNP	Li et al., 2021
JMTV	*A. testudinarium, I. sinensis, Hae. longicornis*	Tick	China	2018–2019	OM459837–OM459849	Niu et al., 2022 *
JMTV^6^	*Homo sapiens*	Human	Russia	2016	MN218697–MN218698	Ternovoi et al., 2020a *

ALSV	*Homo sapiens*	Human	China	2017	MH158415–MH158438	Wang et al., 2019a
ALSV	*I. persulcatus*	Tick	China	2017	MH158439–MH158440, MH678646–MH678648	
ALSV	*An. yatsushiroensis, Ae. vexans, Cx. pipiens pallens, Cx. tritaeniorhynchus*	Mosquito	China	2017	MK213942, UNP	
ALSV	*I. ricinus*	Tick	Finland	2011, 2017	MN107153–MN107160	Kuivanen et al., 2019
ALSV	*Ovis aries*	Sheep	China	2017	MK122719, MK122721, MN218596, MN218597	Wang et al., 2019b
ALSV	*Bos taurus*	Cattle	China	2017	MK122718, MK122720, MN218594, MN218595	
ALSV^7^	*I. ricinus*	Tick	France	UNK	MN095519-MN095522	Temmam et al., 2019
ALSV	*I. persulcatus, D. nuttalli, Hae. concinna, I. ricinus, D. reticulatus*	Tick	Russia	2012–2019	MN604229, MN648770–MN648777, MT210218–MT210225, MW525311, MW525312, MW525314–MW525321, MW52531–MW52533, MW525284–MW525310, MW556738–MW556741, MW584331	Kholodilov et al., 2020
ALSV	*I. ricinus*	Tick	Serbia	2016	MT822179–MT822180	Stanojević et al., 2020
ALSV	*I. persulcatus*	Tick	China	2018	MT246198–MT246199, MT514916–MT514917, MT536950–MT536953	Cai, 2020 *
ALSV^8^	*I. species*	Tick	Germany	2019–2020	MW094132–MW094159	Boelke et al., 2020 *
ALSV^8^	*Cervus elaphus*	Deer	Germany	2019–2020	UNP	
ASLV^9^	*Hae. Longicornis*	Tick	China	UNK	MZ676705	Hu, 2022 *

YGTV	*D. nuttalli, D. marginatus*	Tick	Russia	2014–2016	MW525322–MW525325, MW556730–MW556737	Kholodilov et al., 2021
YGTV	*D. nuttalli, Unspecified tick* spp.	Tick	China	2014–2017	MH688529–MH688539, MH688679–MH688696, MT248418–MT248421	Shen et al., 2019; Shi J. M., 2021*

TAKV	*Hae. formosensis*	Tick	Japan	2019–2020	LC628180–LC628199	Kobayashi et al., 2021

NTV^10^	*I. holocyclus*	Tick	Australia	2019–2020	OK128264	Gofton et al., 2022

XJTV1	UNK	Tick	China	2017–2018	MZ244282–MZ244285	Yang and Zhang, 2021 *

PLJV^7^	*Pteropus lylei*	Bat	Cambodia	2015–2016	MN095531–MN095534	Temmam et al., 2019

FLSV	*Peromyscus leucopus*	Rodent	USA	2014	MN811583–MN811584	Vandegrift et al., 2020

GDJLV	UNK	Cattle feces	China	2017	MW896893–MW896896	Chen et al., 2022 *

HJLV	UNK	Soil	China	2018	MW896920–MW896923	Chen et al., 2022 *

JMTV strains referred to as ^1^Mogiana tick virus; ^2^Guangxi tick virus or Amblyomma virus; ^3^Centeno and Aripo Savannah; ^4^Kindia tick virus; ^5^Rimini; ^6^Manych virus; ^7^Sequence referred to as JMTV; ALSV strain referred to as ^8^Harz mountain virus; ^9^Liaoning. ^10^Newport Tick virus, also referred to as Ixodes holocyclus jingmenvirus *Unpublished data, information found on Genbank. Ref., reference; UNP, unpublished sequence; UNK, unknown; JMTV, Jingmen tick virus; ALSV, Alongshan virus; YGTV, Yanggou tick virus; TAKV, Takachi virus; NTV, Newport Tick virus; XJTV1, Xinjiang tick virus 1; PLJV, Pteropus lylei jingmenvirus; FLSV, Flavi-like segmented virus strain US001; GDJLV, Guangdong jingmen-like virus; HJLV, Hainan jingmen-like virus. *R., Rhipicephalus; Hae., Haemaphysalis; I., Ixodes; D., Dermacentor; A., Amblyomma; Hya., Hyalomma; Ae., Aedes; An., Anopheles; Cx., Culex; Rat., Rattus; Apo., Apodemus; Mic., Microtus; Cri., Cricetulus; Rho., Rhombomys; Mer., Meriones; Myo., Myotis; Min., Miniopterus; Nyc., Nyctalus; Pip., Pipistrellus; Ept., Eptesicus; Rhi., Rhinolophus.*

In addition to arthropods, JMTV RNA was also detected in vertebrates, either in mammals-derived samples such as cattle serum (Qin et al., 2014; Souza et al., 2018; Guo J. J. et al., 2020), primate plasma (Ladner et al., 2016), serum and organs from 11 species of bats from 6 genera and 11 species of rodents from 7 genera (Guo J. J. et al., 2020; Yu et al., 2020); or reptile-derived samples such as tortoise blood (Ogola et al., 2022). Finally, JMTV genomes were recovered from three acute phase serum samples collected from Crimean-Congo hemorrhagic fever orthonairovirus patients as well as from skin biopsies from four patients with tick bites (Emmerich et al., 2018; Jia et al., 2019). While the reported prevalence of JMTV RNA in vertebrates is lower than in ticks, it is still remarkable for cattle from China (3.5–10%) and Brazil (14%), for bats (12%) and rodents (7%) from China and for tortoises from Kenya (67%) (Qin et al., 2014; Souza et al., 2018; Guo J. J. et al., 2020; Yu et al., 2020; Shi et al., 2021; Ogola et al., 2022). Moreover, an in-depth study of rodents from China detected JMTV in all tested organs (liver, kidney, lung, heart, and spleen), preferentially in the liver samples (25%), and particularly in *Apodemus uralensis* (29%) and *Microtus arvalis* (24%) (Yu et al., 2020).

In concordance with RNA detection, anti-JMTV antibodies have been found in cattle from three regions of China with 18–37% seroprevalence (Qin et al., 2014; Shi et al., 2021). Moreover, patients seronegative for tick-borne encephalitis virus (TBEV) from China with a history of tick bites were tested for seroconversion to JMTV and 1.6% were positive (Jia et al., 2019). Furthermore, one sporadic detection of anti-JMTV antibodies was reported in a cohort of 70 patients with recorded tick bites from France (Temmam et al., 2019). Taken together with the RNA detections listed above, these data strongly suggest that JMTV could replicate in vertebrates, notably cattle and humans, although the tropism, pathogenesis, and clinical manifestations of the infection remain unknown.

**FIGURE 4:**
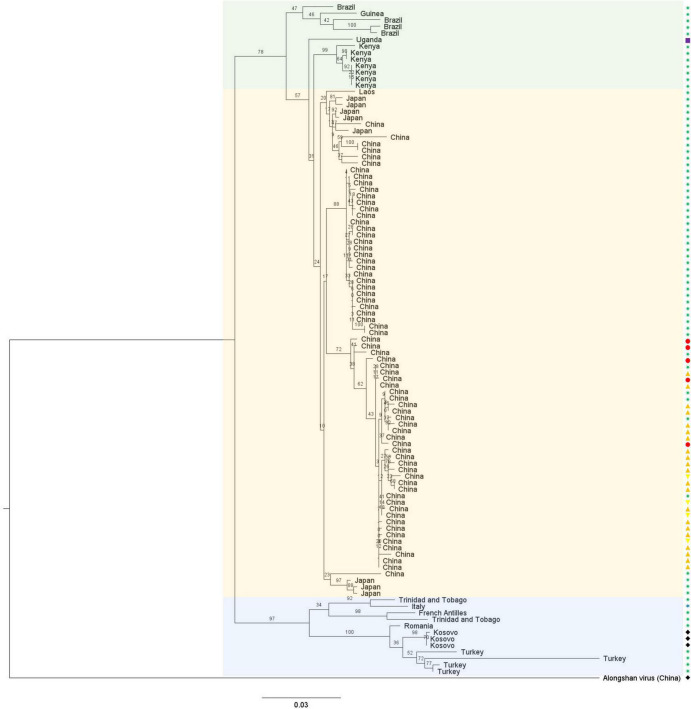
Phylogenetic analysis of all published full-length NSP1 ORF amino acid sequences of JMTV strains, and the prototype strain of ALSV as an outgroup. This tree was built with PhyML with an LG substitution model and midpoint-rooted. The branches are labeled with the bootstrap proportion in percentages (out of 100 bootstraps), the tips are labeled with the sample collection location, and the bar represents 0.03 substitutions per nucleotide position. JMTV strains seem to cluster according to their detection location rather than host with two clear subclades, the European and Central American strains (shaded in blue) on one side and the Asian (shaded yellow) and African and South American (shaded green) strains on the other side. The hosts are represented with symbols on the side of the phylogenetic tree: ticks are green stars with eight branches, mosquitoes are blue stars with six branches, monkeys are purple squares, cattle are red circles, bats are orange triangles pointing up, rodents are yellow triangles pointing down and humans are black diamonds.

To date, only limited ecological data relate to the transmission cycle of this virus. Indeed, JMTV was detected in adult ticks, both males and females, as well as in nymphs and unfed larvae, suggesting it could be vertically transmitted (Maruyama et al., 2014; Jia et al., 2019; Pascoal et al., 2019; Kobayashi et al., 2021). It has also been detected in the salivary glands of naturally and experimentally infected male ticks, suggesting the virus could be transmitted through an infected tick bite (Maruyama et al., 2014; Jia et al., 2019). Furthermore, the analysis of the codon usage bias of JMTV supports the hypothesis that it is a true arbovirus, cycling between an arthropod vector and a vertebrate host (Maruyama et al., 2014). Finally, sequence analysis revealed that JMTV genetic diversity is more strongly influenced by geographic distance than by the host from which the sequence originated, which is compatible with a vector-borne transmission cycle (Figure 4; Guo J. J. et al., 2020).

Despite a strong presence in environmental samples, the virus does not seem to be fit for replication in the laboratory models tested to date. Indeed, most authors report that isolating the strains they sequenced was unsuccessful, or could only be sustained for a couple of passages, on BME26 cells (*R. microplus*, tick), C6/36 cells (*Aedes albopictus*, mosquito), DH82 cells (*Canis familiaris*, dog) or Vero E6 cells (*Cercopithecus aethiops*, monkey) at low titers and did not necessarily produce cytopathic effect (CPE) (Maruyama et al., 2014; Qin et al., 2014; Ladner et al., 2016; Souza et al., 2018; Dinçer et al., 2019; Meng et al., 2019; Kobayashi et al., 2021; Shi et al., 2021; Ogola et al., 2022). Jia et al. (2019) reported sustained JMTV replication in the BME/CTVM23 (*R. microplus*) cell line with a 3-log titer increase by day 7, but the number of positive passages is not mentioned. These limited models enabled the observation of thin sections of infected DH-82 cells and their negatively stained supernatant by transmission electron microscopy which suggested that JMTV particles are enveloped and slightly larger than flaviviruses, with a diameter of 70–80 nm and clear protrusions (Qin et al., 2014). Other, unsuccessful, laboratory models tested were the following cells lines: BHK-21 (*Mesocricetus auratus*, hamster), LLC-PK1 (*Sus scrofa*, pig), PK-15 (*Sus scrofa*, pig), MDBK (*Bos taurus*, bovine), Vero (*Cercopithecus aethiops*, monkey), HEK-293 (*Homo sapiens*, human), DF-1 (*Gallus gallus*, chicken), and new-born mice (Maruyama et al., 2014; Souza et al., 2018; Meng et al., 2019; Shi et al., 2021). Interestingly, JMTV RNA was reported to be detected in midguts and salivary glands of experimentally infected adult male *A. javanense*, which suggests live tick models could provide more sustainable and consistent laboratory models than cell culture (Jia et al., 2019). Setting up a laboratory model fitting JMTV replication would be a clear step toward a better fundamental understanding of this virus. Finding and studying virus strains or species that are better suited for replication *in vitro* could be another solution.

Since 2014, segmented flavi-like virus sequences have been detected worldwide. Some of these sequences share over 95% identity with JMTV (Kindia tick virus, Guangxi tick virus, Amblyomma virus, or Manych virus) and are therefore likely to all belong to the same species (Jia et al., 2019; Ternovoi et al., 2020b; Xu et al., 2020). Sequences from novel segmented flavi-like virus species have also been identified and grouped under the putative genus name *Jingmenvirus*.

### Alongshan virus

A novel jingmenvirus tentatively named Alongshan virus (ALSV) was isolated and sequenced from a blood sample taken from a human patient reporting tick bites in China in 2017 (Wang et al., 2019a). The genome organization of ALSV is similar to that of JMTV, except for the fact that segment 2 seems to be at least bicistronic, with two overlapping reading frames VP1a and VP1b, as well as the putative open reading frame nuORF (Figure 1; Wang et al., 2019a). ALSV NSP1 and NSP2 share approximately 80% amino acid similarity with JMTV, the segments are 3 prime polyadenylated, and the segment termini conserved sequences are homologous between the two viruses. The structural proteins share only 25–75% amino acid similarity with their JMTV counterparts, suggesting that ALSV is a novel species in the *Jingmenvirus* genus (Wang et al., 2019a).

Similarly to JMTV, ALSV was subsequently detected in a number of tick and mosquito hosts in varying prevalence, *I. persulcatus, I. ricinus, D. nuttalli, D. reticulatus, Hae. concinna, Hae. longicornis, Anopheles yatsushiroensis, Ae. vexans, Cx. pipiens pallens*, and *Cx. tritaeniorhynchus*, as well as from sheep, cattle, and deer sera, from locations in Eurasia: in China, Finland, Russia, Serbia, Germany, and France (Kuivanen et al., 2019; Temmam et al., 2019; Wang et al., 2019a,b; Boelke et al., 2020; Kholodilov et al., 2020, 2021; Stanojević et al., 2020; Hu, 2022) (Table 1 and Figure 3). Its main host seems to be *Ixodes* ticks, which are the main vector for TBEV transmission in Europe (Jääskeläinen et al., 2011; European Centre for Disease Prevention and Control [ECDC], and European Food Safety Authority [EFSA], 2018, 2020; Chitimia-Dobler et al., 2019). *Ixodes* ticks are widely distributed in Asia and Europe, and common hosts include sheep, cattle, horses, dogs, rabbits, and humans (Jääskeläinen et al., 2011; European Centre for Disease Prevention and Control [ECDC], and European Food Safety Authority [EFSA], 2018, 2020; Chitimia-Dobler et al., 2019).

In the north-eastern region of China, 9% of sheep and 5% of cattle were found to have ALSV-reactive antibodies, and 4% of sheep and 2% of cattle had neutralizing antibodies (Wang et al., 2019b). Moreover, a retrospective survey of patients who presented with undiagnosed symptoms and a history of tick bites in the same region as the prototype found evidence of ALSV in 23% of around one hundred tested samples (Wang et al., 2019a). The patients had non-specific clinical symptoms (headache and fever) and all had a complete recovery (Wang et al., 2019a). Seroconversion and seroneutralization against ALSV were detected in all tested positive patients, which, according to the authors, suggests the induction of a humoral immune response (Wang et al., 2019a).

The detection of JMTV and ALSV in human samples is notable, particularly considering that their tropism and pathogenesis remain to be formally elucidated. This could be facilitated by the fact that ALSV seems to be better suited to classical laboratory models than JMTV. Indeed, the virus was isolated from human, cattle, and sheep samples from China and passaged in Vero cells by Wang et al., which produced CPE at earlier time points at every passage, suggesting a certain level of cell adaptation (Wang et al., 2019a,b). However, Kuivanen et al. (2019) have found that their Finnish ALSV isolates did not replicate on Vero cells. Limited ALSV replication was found in a range of human cell lines (SH-SY5Y, WISH, SMMC, THP-1) after inoculation with a concentrated stock of virus (Wang et al., 2019a). Kholodilov et al. (2020, 2021) found that two tick cell lines (IRE/CTVM19 from *I. ricinus* and HAE/CTVM8 from *Hya. anatolicum*) can successfully sustain persistent infection by a Russian strain of ALSV, over up to several years, with no CPE. Balb/c mice were inoculated intraperitoneally with cell culture supernatant containing a Chinese strain of virus, with 10^7^ copies of RNA and were found to present pathological changes in the liver, kidneys, and brain 14 days post-injection (Wang et al., 2019a). Viral RNA was detected in the liver, spleen, and lung tissues (10^7^ RNA copies/mL), as well as in the kidney and heart tissues (10^6^ RNA copies/mL) but to a lesser extent in brain tissues and blood (10^4^ RNA copies/mL) 30 days post-infection (Wang et al., 2019a). However, ALSV replication was not detected in BHK-21, Hepa 1-6 (*Mus musculus*, mouse), or in U-87MG, HFF, Caco-2, SK-N-SH, and CRL-2088 (*Homo sapiens*, human) cell lines (Kuivanen et al., 2019; Wang et al., 2019a).

This limited replication in laboratory models enabled the imaging of ALSV particles, by transmission electron microscopy of thin sections of infected Vero cells and negatively stained purified particles. The prototype ALSV particles, from China, are enveloped and larger than flaviviruses, with a diameter of 80–100 nm (Wang et al., 2019a). However, Kholodilov et al. (2020) reported that purified ALSV particles from a Russian isolate had a diameter of around 40 nm, accompanied by smaller particles, with a diameter of 13 nm. It is not clear why such a difference in size was observed between these Chinese and Russian isolates of ALSV, but this discrepancy highlights the need for a stable and widely available laboratory replication system for jingmenviruses.

Some ALSV proteins have been characterized despite the lack of a stable replication model. Pending X-ray crystallography and cryo-electron microscopy confirmation, Garry and Garry have used structural models to show that the jingmenvirus glycoproteins share similarities with the glycoproteins of flaviviruses, alphaviruses and bunyaviruses, termed class II viral fusion proteins (Garry and Garry, 2020). The putative fusion peptide loop was identified in ALSV VP1a (amino acids 119–129), and one of the subdomains of VP1a shows high structural similarities with the flaviviral E domain III (Garry and Garry, 2020). An additional, jingmenvirus-specific domain was predicted, as a mucin-like domain which is thought to be modified by *O*-glycosylation(s). Such post-translational modifications may shield the virus from recognition by the host’s immune system (Garry and Garry, 2020). Despite the difference in organization of the segment coding for the glycoprotein, their models applied to both JMTV VP1 and ALSV VP1a, but not to insect-associated jingmenviruses (see below) (Garry and Garry, 2020).

Besides the models built for the structural proteins, a recombinant version of the ALSV protein NSP2 was used to determine a 2.9 Å-resolution structure of its helicase domain by X-ray crystallography (PDB 6M40) (Figure 2; Gao et al., 2020). The ATPase activity of the recombinant protein was confirmed and it was shown that structural features at the ATPase active site and RNA-binding groove remain conserved between flaviviruses and ALSV (Gao et al., 2020).

### Other tick-associated jingmenviruses

Only four other tick-associated jingmenviruses species have been reported to date (Table 1). Yanggou tick virus (YGTV) was first identified from ticks collected in China in 2014 [unpublished data, deposited on Genbank by Shi J. M. (2021)]. Viral sequences were identified from 21 samples in that original collection, 3 from *D. nuttalli* and 18 from unspecified tick species. YGTV RNA was subsequently detected in two *D. marginatus* and one *D. nuttalli* samples during a tick survey in Russia during the period 2011-2019. Although the number of samples remains low, YGTV seems to be associated to *Dermacentor spp.* ticks (Kholodilov et al., 2021). Its genome organization resembles that of ALSV, with VP1a and VP1b in segment 2. YGTV shares 80% amino acid similarity over NSP1 and NSP2 with both JMTV and ALSV, and 50–60% amino acid similarity over the structural proteins. Similarly to ALSV, YGTV positive samples were used in a successful isolation attempt on IRE/CTVM19 and HAE/CTVM8 tick cells, with a persistent infection displaying no CPE (Kholodilov et al., 2021).

Takachi virus was detected in five pools of *Hae. formosensis* nymphs collected by flagging in Japan between 2019 and 2020. The virus could not be isolated on Vero or BHK-21 cells, but its genome sequence was determined and shares 55–85% amino acid similarity with JMTV and ALSV (Kobayashi et al., 2021).

**FIGURE 5:**
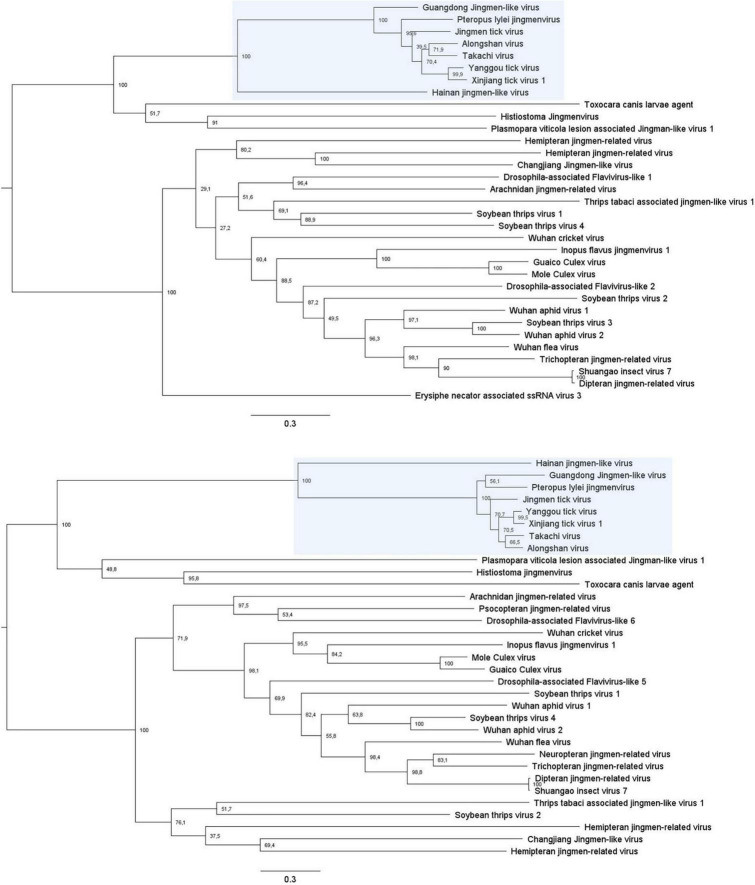
Phylogenetic analysis of all known jingmenvirus species with full-length NSP1 **(Top)** and NSP2 **(Bottom)** ORF amino acid sequences. These trees were built with PhyML with an LG substitution model and midpoint rooted. The branches are labeled with the bootstrap proportion in percentages (out of 1000 bootstraps) and the bars represent 0.3 substitutions per nucleotide position. Jingmenviruses cluster in two main clades, on the one hand the tick- and vertebrate-associated jingmenviruses (shaded in blue) and on the other hand, insect-associated jingmenviruses.

Newport Tick virus (also named Ixodes holocyclus jingmenvirus) was identified in a metagenomics study of ticks from New South Wales in Australia (Gofton et al., 2022). The sequences were detected in relatively high prevalence in *I. holocylcus* ticks from Kioloa and Sydney. According to the authors, the largest contig (2087 nt) encodes the complete putative glycoprotein, which shares approximately 62% homology to JMTV, ALSV, and YGTV (from BLAST results). A MUSCLE alignment using the available glycoprotein sequences of tick- and vertebrate-associated jingmenviruses shows that this sequence shares between 51 and 55% homology with one group of tick- and vertebrate-associated jingmenviruses (ALSV, YGTV, XJTV1, TAKV) and between 27 and 30% with the other group (JMTV, PLJV and GDJLV – see below). This virus could not be included in our phylogenies (Figure 5) since no NSP1 or NSP2 sequences have been published yet. Additional sequences from other segments would be needed to confirm the segmented nature of this virus genome and its position in the phylogenies.

Finally, Xinjiang tick virus 1 was detected from unspecified ticks from China [unpublished data, deposited on Genbank by Shi J. M. (2021)]. At this time, no additional information is available on these sequences. An analysis using NCBI BLASTx shows that the virus with the most similar sequences is YGTV and pairwise MUSCLE alignments show that their ORFs share 75–91% amino acid similarity (National Library Medicine [NIH], 2022).

### Jingmenvirus sequences related to tick-associated jingmenviruses

A complete coding sequence for a novel segmented flavi-like virus was detected in urine specimens from *Pteropus lylei* bats from Cambodia (Table 1; Temmam et al., 2019). The viral sequences share 70–80% similarity with JMTV and ALSV over the non-structural proteins NSP1 and NSP2 and 40–55% similarity over proteins encoded by segments 2 and 4. The genome organization is similar to that of JMTV with a single VP1 ORF in segment 2 (Figure 1).

A partial novel jingmenvirus sequence was detected in serum samples collected from *Peromyscus leucopus* mice in the USA in 2011–2017 (Vandegrift et al., 2020). These sequences were not included in our phylogenies (Figure 5) since they are partial sequences, covering part of NSP2 and most of NSP1, but an alignment showed that these sequences shares under 70% amino acid similarity with JMTV and ALSV.

Two full genome sequences have been identified by metatranscriptomics of environmental samples, namely cattle feces and soil from China (Chen et al., 2022). They have been putatively named Guangdong jingmen-like virus and Hainan jingmen-like virus, respectively. Their host remains to be identified but the presence of jingmenvirus sequences in the environment is another proof that these viruses are ubiquitous and need to be thoroughly characterized.

These jingmenvirus sequences detected from mammals and the environment cluster with the tick-associated jingmenviruses in both non-structural protein-derived phylogenies (Figure 5), which could suggest these viruses follow a classical arbovirus horizontal transmission cycle (Colmant et al., 2022). The optimal models for the phylogenies presented here were selected using the server Smart Model Selection in PhyML (Lefort et al., 2017).

## Insect- and other host-associated jingmenviruses

Sequences for 40 other putative segmented flavi-like virus species have been detected from a range of hosts, as described below and listed in Table 2. As mentioned above, the sequences seem to phylogenetically cluster in two separate groups (Figure 5), with, broadly, on the one hand the tick- and vertebrate-associated jingmenviruses, and on the other hand the insect-associated jingmenviruses, in a way that is remindful of vertebrate-infecting and insect-specific flaviviruses being separated in separate clades (Blitvich and Firth, 2015; Vandegrift et al., 2020; Zhang et al., 2020).

**TABLE 2:** Insect- and other host-associated jingmenviruses RNA detections.

Virus	Host species	Host type	Location	Date	Genbank number	References
Guaico Culex virus	*Cx. interrogator; Cx. coronator*	Mosquito	Panama	2012	KM521552–KM521560; KM521566–KM521570	Ladner et al., 2016
	*Cx. coronator*		Peru	2009	KM521561–KM521565, KM461666–KM461670	
	*Cx. declarator*		Trinidad	2008	KM521571–KM521574	
	*Cx.* spp.		Brazil	2010	KT966498–KT966501, KX762047	Pauvolid-Corrêa et al., 2016
Mole Culex virus	*Cx.* spp.	Mosquito	Ghana	2016	LC505052–LC505055	Amoa-Bosompem et al., 2020
Charvil virus	*Drosophila ananassae*, *Drosophila melanogaster*, *Drosophila malerkotliana, Scaptodrosophila latifasciaeformis*	Drosophila	UK	2010	KP714089	Webster et al., 2015
Drosophila-associated Flavivirus-like 1			Kenya	2010	KP757923	
Drosophila-associated Flavivirus-like 2			Kenya	2010	KP757924	
Drosophila-associated Flavivirus-like 5			UNK	UNK	KP757926	
Drosophila-associated Flavivirus-like 6			UNK	UNK	KP757927	
Shuangao insect virus 7^1^	*Chrysopidae* sp., *Psychoda alternata, Diptera* sp.	Insect	China	2013	KR902717–KR902720	Shi et al., 2016b
	*Clogmia albipunctata*	Fly	USA	2012	MW314686–MW314689	Paraskevopoulou et al., 2021
Wuhan flea virus	*Ctenocephalides felis*	Flea	China	2013	KR902713–KR902716	Shi et al., 2016b
Ctenocephalides felis flavi-like virus^2^	*Ctenocephalides felis*	Flea	USA	2012	MW208795, MW208800	Wu et al., 2020; Paraskevopoulou et al., 2021
Wuhan aphid virus 1	*Hyalopterus pruni*	Aphid	China	2013	KR902721–KR902724	Shi et al., 2016b
	*Neohydatothrips variabilis*	Thrips	USA	2018	MW023847–MW023850	Thekke-Veetil et al., 2020
	*Rhopalosiphum maidis, Rhopalosiphum padi, Sitobion avenae*	Aphid	Japan	2016–2018	LC516839–LC516842	Kondo et al., 2020
Wuhan aphid virus 2	*Hyalopterus pruni, Aulacorthum magnoliae*	Aphid	China	2013	KR902725–KR902728	Shi et al., 2016b
	*Pisum sativum*	Peas	France	2011	MK948535–MK948538	Gaafar and Ziebell, 2020
Wuhan cricket virus	*Conocephalus* sp.	Crickets	China	2013	KR902709–KR902712	Shi et al., 2016b
Culicoides jingmenvirus^3^	*Culicoides* sp.	Biting midge	Senegal	2013	UNK	Temmam et al., 2016
Changjiang Jingmen-like virus	*Procambarus clarkii*	Crayfish	China	2014	KX883002–KX883003	Shi et al., 2016a
Trichopria drosophilae flavi-like virus	*Trichopria drosophilae*	Drosophila	France	2012	UNK	Wu et al., 2020
Aphalara polygoni flavi-like virus 1	*Aphalara polygoni*	Insect	UK	2013		
Ischnodemus falicus flavi-like virus	*Ischnodemus falicus*	Insect	USA	2013		
Trioza urticae flavi-like virus	*Trioza urticae*	Insect	UK	2013		
Ulopa reticulata flavi-like virus	*Ulopa reticulata*	Insect	Germany	2012		
Heterocaecilius solocipennis flavi-like virus^4^	*Heterocaecilius solocipennis*	Insect	Japan	2012	MW208798, MW208804	Wu et al., 2020
Plea minutissima flavi-like virus^5^	*Plea minutissima*	Insect	Germany	2011	MW208797, MW208803	
Tachycixius pilosus flavi-like virus^6^	*Tachycixius pilosus*	Insect	Germany	2012	MW208796, MW208802	
Soybean thrips virus 1	*Neohydatothrips variabilis*	Thrips	USA	2018	MW023851–MW023853	Thekke-Veetil et al., 2020
Soybean thrips virus 2					MW023854–MW023857	
Soybean thrips virus 3					MW033628–MW033631	
Soybean thrips virus 4					MW033625–MW033627	
Soybean thrips virus 5					MW033632	
Carajing virus	*Culicoides arakawae*	Biting midge	Japan	2017	LC552035–LC552038	Kobayashi et al., 2020
Arachnidan jingmen-related virus OKIAV333	*Euscorpius sicanus*	Scorpion	Italy	2012	MW314684–MW314685	Paraskevopoulou et al., 2021
Trichopteran jingmenvirus OKIAV337	*Costora delora*	Insect	Australia	2013	MW314690–MW314693	Paraskevopoulou et al., 2021
Dipluran jingmen-related virus OKIAV326	*Campodea silvestrii*	Insect	Germany	2012	MW314694	
Neuropteran jingmenvirus OKIAV339	*Pseudomallada ventralis*	Insect	Austria	2012	MW208799, MW208801, MW208805, MW208806	
Thrips tabaci associated jingmen-like virus 1	*Thrips tabaci*	Thrips	Italy	2018	MN764158–MN764159	Chiapello et al., 2021
Histiostoma Jingmenvirus	*Histiostoma* sp.	Mite	Germany	2014	MT747997–MT748000	Guo L. et al., 2020
Inopus flavus jingmenvirus 1	*Inopus flavus*	Flies	Australia	2019	OM869459–OM869462	Colmant et al., 2022
Toxocara canis larvae agent	*Toxocara canis*	Nematode	Scotland	UNK	EU792509, EU792511	Tetteh et al., 1999; Callister et al., 2008
Plasmopara viticola lesion associated Jingmen-like virus 1	*Plasmopara viticola*	Fungus	Italy	2018	MN551114, MN551116	Chiapello et al., 2020
Erysiphe necator associated ssRNA virus 3	*Erysiphe necator*	Fungus	Italy	2018	MN558700	Rodriguez-Romero et al., 2021 *

UNK, Unknown. UK, United Kingdom; USA, United States of America. Cx., Culex. *Unpublished data, information found on Genbank. Also referred to as ^1^Dipteran jingmenvirus OKIAV332; ^2^Siphonapteran jingmen-related virus OKIAV340 (>90% amino acid identity to Wuhan flea virus); ^3^STE0043 contig 1088; ^4^Psocodean jingmen-related virus OKIAV331; ^5^Hemipteran jingmen-related virus OKIAV327; ^6^Hemipteran jingmen-related virus OKIAV329.

### Mosquito-associated jingmenviruses

Jingmenviruses have also been found in other arthropods, including mosquitoes. The most studied insect-associated jingmenvirus is Guaico Culex virus (GCXV), which was isolated from six *Culex* mosquito pools (*Cx. declarator, Cx. Coronator*, and *Cx. interrogator*) collected from Trinidad, Peru, and Panama between 2008 and 2013, and subsequently from two *Culex* spp. mosquito pools collected from Brazil in 2010 (Ladner et al., 2016; Pauvolid-Corrêa et al., 2016; Table 2). The genome sequence was shown to include four to five non-polyadenylated segments, depending on the isolate, comprising conserved termini sequences (Ladner et al., 2016). Segment 1 codes for NSP1, related to the flaviviral NS5, segment 2 codes for NSP2, related to the flaviviral NS3, while segments 3 and 4 each code for three ORFs, most likely structural proteins (VP1 to VP6), and segment 5 codes VP7 (Figures 1, 2; Ladner et al., 2016). Zhang et al. (2021) have confirmed that GCXV NSP2 is an RNA helicase that can unwind RNA structures in both 3 prime and 5 prime directions in an ATP-dependent manner. NSP2 also has an RNA chaperone activity that can remodel structured RNA and facilitate RNA strand annealing, independently of the presence and catalysis of ATP (Zhang et al., 2021).

Viral replication was detected in three mosquito cell lines (C6/36 *Ae. albopictus*, CT *Cx. tarsalis*, Aag2 *Ae. aegypti*) and intrathoracically inoculated female mosquitoes (*Ae. albopictus* and *Cx. quinquefasciatus*), but not in tick (ISE6 *I. scapularis*), sand-fly (LL-5 *Lutzomyia longipalpis*) or vertebrate (Vero, BHK-21, DF-1) cell lines, or in intracranially inoculated new-born mice. No significant vertical transmission was detected in mosquitoes. Ladner et al. (2016) developed a reverse genetics system to show that replication could occur with and without segment 5. The authors demonstrated that, remarkably, each segment can be packaged separately, in a multicomponent viral system. Purified enveloped virions were found to be 30–35 nm in diameter.

Another *Culex*-associated jingmenvirus tentatively named Mole Culex virus (MoCV) was isolated from three pools of *Culex* mosquitoes collected from Ghana in 2016 and caused CPE on C6/36 cells (Amoa-Bosompem et al., 2020). Its genome organization is similar to that of GCXV and the sequences for NSP1 and NSP2 share 80 and 70% amino acid similarity with those of GCXV respectively, while the two viruses share between 50 and 80% amino acid similarity over VP1 to VP6 (VP1: 70%, VP2: 50%, VP3: 70%, VP4: 80%, VP5: 60%, VP6: 70%).

Contrary to their tick-associated counterparts, GCXV and MoCV were found in low prevalence in their arthropod host and there is no evidence of any association with vertebrates.

### Other host-associated jingmenvirus sequences

Sequences for jingmenvirus-related putative viruses have been found in various arthropods including insects such as fleas, flies, aphids, crickets, biting midges, drosophilae, plant lice, thrips, as well as mites, scorpions, and crayfish (Webster et al., 2015; Ladner et al., 2016; Shi et al., 2016a,b; Temmam et al., 2016; Guo L. et al., 2020; Kobayashi et al., 2020; Kondo et al., 2020; Thekke-Veetil et al., 2020; Wu et al., 2020; Chiapello et al., 2021; Paraskevopoulou et al., 2021; Colmant et al., 2022). These “insect-associated” jingmenvirus sequences do not contain poly(A) tails, the second segment is bicistronic (VP4 before VP1, see Figure 1) and was reported to be expressed in much higher numbers than the other three segments, and compared to JMTV (Shi et al., 2016b). None of these have been isolated and all remain putative viruses.

Jingmenvirus-related sequences have also been detected worldwide in a range of non-arthropod- and non-vertebrate-derived samples, including nematodes, fungus, and plants (Table 2 and Figure 3) (Tetteh et al., 1999; Callister et al., 2008; Qin et al., 2014; Chiapello et al., 2020; Gaafar and Ziebell, 2020). The classification of some of these short partial sequences as genomic sequences from putative viruses is purely hypothetical, until more segment sequences are elucidated (Matsumura et al., 2017; Rodriguez-Romero et al., 2021). In particular, Qin et al. (2014) identified jingmenvirus-related sequences in previously published sequencing data, based on similarities with JMTV (Tetteh et al., 1999; Callister et al., 2008). These sequences originating from the nematode *Toxocara canis* were originally attributed to a putative *Toxocara canis* larvae agent (TCLA). Of note is the fact that human patients were found to be seropositive against recombinant proteins from the putative structural ORFs (Tetteh et al., 1999; Callister et al., 2008).

The phylogeny of insect-associated jingmenvirus sequences does not follow the phylogeny of their putative identified insect hosts, which is different from what has been found for insect-specific flaviviruses (Figure 5; Colmant et al., 2017, 2022). This is corroborated by the presence of crayfish-, scorpion-, fungus- and plant-associated jingmenvirus sequences within the phylogeny. This suggests these putative viruses have not co-evolved with their hosts, which could be due to multiple reasons. The hosts could have been incorrectly assigned, due to data originating from metagenomics studies, which cannot differentiate sequences from an insect or from its previous meal, or a contaminating parasite or fungus (Colmant et al., 2022). Jingmenviruses could also exist in non-arthropod reservoirs, such as plants or fungi, which would allow independent evolution. Remarkably, the complete genome sequence of putative virus Wuhan aphid virus 2, originally detected in two aphid species in China, was detected in peas (*Pisum sativum*) from France, Germany, and Austria (Shi et al., 2016b; Gaafar and Ziebell, 2020). The novel sequences share between 90 and 97% amino acid identity with the prototype strain, depending on the segment (Gaafar and Ziebell, 2020). This close relationship could suggest that the virus can be transmitted horizontally between aphids and plants. This would support the lack of co-evolution between insect and insect-associated jingmenvirus sequence phylogenies.

## Discussion

Taken together, these data clearly show that the vast majority of sequences published are identified as putative jingmenvirus genomes rather than sequences from isolated jingmenviruses. While metagenomics provides essential information on sequence diversity, these efforts should be combined with classical virology to identify and characterize the putative viruses, their hosts, and the interactions between the two. Indeed, Taniguchi wrote in 2019 that “the establishment of JMTV cultivating systems in mammalian cell lines and of a reverse genetics system is the next challenge” to study JMTV, but these milestones have not been reached to date (Taniguchi, 2019). The mechanisms involved in jingmenvirus replication restriction in laboratory models are not yet clear. It was shown that Inopus flavus jingmenvirus 1 can elicit an immune response from its host via the RNA interference pathways (Colmant et al., 2022). However, a non-permissive cell line tested for this virus has a dysfunctional RNAi response, so this mechanism is unlikely to be preventing virus replication *in vitro* (Brackney et al., 2010).

A stable laboratory replication model for jingmenviruses will be essential to investigate the viral tropism, replication mechanisms, host restriction, and potential pathogenicity in different hosts. The elucidation of the 3D structures, in particular that of the variable structural proteins, would provide insights into the mechanisms involved in binding and entry into host cells, potential immune response evasion mechanisms, and contribute to the development of vaccines and therapies in the context of emergence preparedness. Reverse genetics systems would enable functional studies and help elucidate replication mechanisms as well as reassortment potential. Once these are tools in place, it would also be possible to perform more applied and translational research to uncover potential biotechnological applications of these divergent flavi-like viruses, as it was seen for insect-specific flaviviruses (Hobson-Peters et al., 2019; Hardy et al., 2021; Harrison et al., 2021). In addition to the mechanistic and evolutionary aspects, the technologies mentioned above will be necessary to understand whether jingmenviruses can be responsible for febrile illnesses as it has been reported by some (Emmerich et al., 2018; Jia et al., 2019; Wang et al., 2019a), by fulfilling (virus-adapted) Koch’s postulates (Rivers, 1937; Antonelli and Cutler, 2016).

The mode of transmission of jingmenviruses is another complex aspect of their fundamental characterization that remains to be formally demonstrated. Indeed, jingmenviruses are associated with a range of different hosts. To date, it is considered that jingmenviruses fall into two main clades, insect-associated *versus* tick- and vertebrate-associated jingmenviruses. The first subgroup includes sequences detected in insects, but also in other arthropods such as scorpions or crayfish, and in plants. The mode of transmission of these putative viruses is unknown so far and could vary wildly from one host to another. It would be interesting to establish whether vertical transmission plays a part, as it was seen for insect-specific flaviviruses (Lutomiah et al., 2007; Bolling et al., 2012; Hall-Mendelin et al., 2016; Auguste et al., 2021; McLean et al., 2021; Peinado et al., 2022). Interestingly, some suggest that the transmission could occur horizontally between insects and plants, even though this remains to be demonstrated. This route of transmission has also been hypothesized for a number of insect viruses from other families, and some plant viruses are known to be transmitted by insects (Cui and Holmes, 2012; Vasilakis et al., 2013; Whitfield et al., 2015; Dietzgen et al., 2016; Dáder et al., 2017; Nunes et al., 2017; Newton et al., 2020).

In the case of tick- and vertebrate-associated jingmenviruses, the main hypothesis seems to be horizontal transmission with a tick vector feeding on a vertebrate host, since the same viruses have been found in both types of samples. However, this has not been demonstrated experimentally. Moreover, horizontal transmission vectored by ticks can be very complex, in particular due to the fact that different tick species can have different types of lifecycle. For example, *R. microplus* have a monophasic life cycle, which means that they feed as larvae, molt, feed as nymphs, molt, and feed as adults on a single host individual (Leal et al., 2018). In contrast, *I. persulcatus* have a triphasic life cycle, where they feed on a host as a larva, drop to the ground and molt, seek a new host as nymphs, drop to the ground and molt, and seek a new host again as adults to feed for reproduction (Grigoryeva and Stanyukovich, 2016). We must elucidate the stages at which virus transmission can occur from the tick vector to the vertebrate host. It should be noted that while JMTV and ALSV are mainly associated with *R. microplus* and *I. persulcatus*, respectively, their RNA has been detected in a multitude of other tick species and genera, suggesting that they could replicate in and/or be transmitted by ticks with all types of life cycles. The differences in vector lifecycle could, however, result in differences in transmission efficiency.

In addition to the transmission occurring from a tick vector to a vertebrate host, it is essential to study the transmission routes from ticks to ticks. Indeed, the tick-borne flavivirus TBEV can be transmitted from ticks to ticks horizontally by co-feeding on a non-viremic host, or *via* the sexual route, or vertically *via* the transovarial route, and is known to be transmitted transtadially from one lifecycle stage to the next (egg, larva, nymph, adult) (Labuda et al., 1993; Nuttall et al., 1994; Danielová et al., 2002; Mansfield et al., 2009; Pettersson et al., 2014). All of these routes of transmission need to be explored for jingmenviruses, first to understand ecology of jingmenviruses and the risk associated with their transmission, but also to elucidate the vector competence of different tick species (Labuda et al., 1993). Studying the transmission cycle of jingmenviruses would also confirm current host assignments, attributed using metagenomics methods, which cannot differentiate between a virus actively infecting an insect or present in its last meal or in a parasiting species.

The discovery of segmented flavi-like viruses was a real breakthrough in virus research as they were the first examples of a segmented genome at least partly derived from an unsegmented genome (Bennett, 2014). The evolutionary costs and benefits of segmentation are poorly understood, and the existence of segmented and unsegmented viruses in the same genus raises questions related to their emergence. Are segmented flavi-like viruses more, or as likely to emerge as pathogens as their unsegmented counterparts? What evolutionary or host-adaptation mechanisms would facilitate their emergence? Could reassortments or recombination events be involved (McDonald et al., 2016; Lucía-Sanz and Manrubia, 2017)? Reassortment happens when different strains or species of segmented viruses exchange genetic material (segments) when co-infecting the same cells to produce virus progeny with novel genome combinations (Vijaykrishna et al., 2015). Recombination does not necessitate the existence of a segmented genome, the genetic exchange takes place during RNA replication that can happen by template switching based on sequence homology (Pérez-Losada et al., 2015). In both cases, the genetic diversity of the replicating viruses is enriched, creating new opportunities for viruses to overcome selective pressures and to adapt to new environments and hosts. In the context of evaluating jingmenvirus potential for emergence, this is particularly relevant, considering that evidence of reassortment and recombination events was found by analyzing phylogenetic clustering of JMTV and ALSV isolates, in all segments, as well as several insect-associated jingmenvirus sequences (Qin et al., 2014; Dinçer et al., 2019; Kholodilov et al., 2020; Paraskevopoulou et al., 2021).

The discovery of GCXV and its characterization as a multicomponent flavi-like virus was a second breakthrough for jingmenvirus-associated virus research since it was the first report of a multicomponent viral system in an animal virus (Attar, 2016). Indeed, GCXV is thought to package each segment individually and to require at least three different particles to form a plaque in infected cells (Ladner et al., 2016). Interestingly, GCXV is the only jingmenvirus shown to be multicomponent, and is one of the only jingmenviruses that has been successfully isolated and cultured stably in laboratory models *in vitro* and *in vivo*. The influence of this characteristic on the host restriction displayed by others jingmenviruses in laboratory models should be evaluated. The fact that GCXV branches within the insect-associated jingmenvirus subgroup suggests that it shares a common ancestor with a number of other jingmenviruses. This could suggest that many other jingmenviruses are multicomponent, but this remains to be formally demonstrated. The current knowledge on the structural organization of jingmenviruses is somewhat contradictory, since JMTV was measured to be 70–80 nm, ALSV was found to be 80–100 nm for the Chinese strain and 40 nm with 13 nm smaller particles for the Russian strain, and GCVX was measured at 30–35 nm (Qin et al., 2014; Ladner et al., 2016; Wang et al., 2019a; Kholodilov et al., 2020). The reason for the discrepancy between these sizes is not yet clear, but it could potentially be linked to the multicomponent nature or not of the viral system. What is clear is that these considerations need to be pursued in order to better understand the replication and transmission of jingmenviruses.

In conclusion, jingmenvirus research should be prioritized research topic as they have the potential to emerge as human or veterinary pathogens. Jingmenviruses are found worldwide, including in tick species that are widespread, in close contact with vertebrates, and known to be vectors for other diseases. They can be associated with ticks and vertebrates and could follow a vector-borne transmission cycle. Tick-associated jingmenviruses have been associated with febrile illness in humans. Finally, they are under-studied and therefore the scientific and clinical communities need better preparedness to face a potential future epidemic.

## Author contributions

AC, BC, and RC: conceptualization and writing – review and editing. AC: investigation and writing – original draft. BC and RC: resources. AC and BC: visualization. All authors contributed to the article and approved the submitted version.
